# Prenatal Effects of Nicotine on Obesity Risks: A Narrative Review

**DOI:** 10.3390/ijerph19159477

**Published:** 2022-08-02

**Authors:** Olivia White, Nicole Roeder, Kenneth Blum, Rina D. Eiden, Panayotis K. Thanos

**Affiliations:** 1 Behavioral Neuropharmacology and Neuroimaging Laboratory on Addictions (BNNLA), Clinical Research Institute on Addictions, Department of Pharmacology and Toxicology, Jacobs School of Medicine and Biomedical Sciences, University at Buffalo, Buffalo, NY 14203, USA; oliviawh@buffalo.edu (O.W.); nroeder@buffalo.edu (N.R.); 2 Department of Psychology, University at Buffalo, Buffalo, NY 14203, USA; 3 Division of Addiction Research, Center for Psychiatry, Medicine & Primary Care (Office of Provost), Western University Health Sciences, Pomona, CA 91766, USA; drd2gene@gmail.com; 4 Department of Psychology, Social Science Research Institute, The Pennsylvania State University, University Park, PA 16801, USA; rde5106@psu.edu

**Keywords:** development, maternal smoking, metabolic disease

## Abstract

Nicotine usage by mothers throughout pregnancy has been observed to relate to numerous deleterious effects in children, especially relating to obesity. Children who have prenatally been exposed to nicotine tend to have lower birth weights, with an elevated risk of becoming overweight throughout development and into their adolescent and adult life. There are numerous theories as to how this occurs: catch-up growth theory, thrifty phenotype theory, neurotransmitter or endocrine imbalances theory, and a more recent examination on the genetic factors relating to obesity risk. In addition to the negative effect on bodyweight and BMI, individuals with obesity may also suffer from numerous comorbidities involving metabolic disease. These may include type 1 and 2 diabetes, high cholesterol levels, and liver disease. Predisposition for obesity with nicotine usage may also be associated with genetic risk alleles for obesity, such as the DRD2 A1 variant. This is important for prenatally nicotine-exposed individuals as an opportunity to provide early prevention and intervention of obesity-related risks.

## 1. Nicotine

### 1.1. Epidemiology of Nicotine Usage and Clinical Significance

Cigarette smoking is the leading cause of preventable death worldwide. In the U.S. alone, 435,000 premature deaths occur every year, a part of as many as 5 million worldwide. Despite the prevalence of cigarette smoking declining in recent years, one in every five Americans still smokes cigarettes. Even with this overall decline, the adolescent age group is seeing an increase in the use of tobacco and electronic nicotine systems in recent years. In high school students from 2011 to 2017, the rate of e-cigarette use increased from 1.5 to 11.7 percent [1,2,3,4,5,6,7,8]. As technology develops and new delivery systems appear on the markets, nicotine and tobacco use is a problem far from being eliminated. Especially concerning is the use of tobacco and nicotine in pregnant women. Approximately 22% of women in the U.S. of childbearing age are considered regular cigarette smokers, with only 45% who report quitting upon learning of a pregnancy. Further studies have reported that approximately 26% of pregnant women continue to smoke nicotine throughout their pregnancy [4,9,10]. Nicotine use and cigarette smoking have been identified as being comorbid with a multitude of public health concerns such as psychiatric disorders, a gateway to other substance use such as marijuana and cocaine, cardiovascular disease, diabetes, and cancer as well as several cognitive effects recorded across the developmental lifespan [1,2,6,7,9,11,12].

Daily usage of nicotine has been seen to have numerous negative side effects that influence cognitive and behavioral functioning. In a study looking at middle-aged, primarily Caucasian males undergoing chronic nicotine exposure via cigarette smoking, results showed that fine motor dexterity, general intelligence, and visuospatial learning and memory were recorded to be of inferior performance compared to a control group of nonsmokers [13]. These detrimental effects of nicotine are not limited to adults. A major concern is that women continue to use nicotine products throughout pregnancy, which can have long-term impacts on their child. Maternal smoking during pregnancy is associated with numerous adverse effects including pregnancy complications and fetal development. Prenatal nicotine exposure has not only been indicated to increase risk for complications at birth, but it has also been estimated that exposure to tobacco smoke leads to reduced fetal growth, increased risk for cleft lip/palate, auditory issues, and behavioral problems such as conduct disorder and ADHD seen later in adolescence [4,9,10,14,15,16,17,18,19,20]. Prenatally exposed offspring also have a higher risk for altered brain structure and function related to nicotine’s effects on neurotransmitter systems during development, a possible explanation for these behavioral issues. Adverse effects may continue throughout growth, including later-in-life metabolic dysfunctions, such as obesity [9,10,14]. The current review aims to investigate the effects of prenatal nicotine exposure on obesity risks and the possible mechanisms behind this association.

### 1.2. Nicotine: Mechanisms of Action in Adulthood

Nicotine has been identified as the main addictive constituent within tobacco due to its rewarding effects within the brain that drive individuals to repeatedly smoke. Associative learning plays a major role in the formation of nicotine addiction. Individuals may associate the lighting of a cigarette, for example, with the cognitive and emotional benefits they will feel from smoking. These may include a greater sense of well-being, feeling more relaxed, and reduced anxiety as well as feeling more alert and focused [2,21]. Individuals may seek to feel these effects again with continued nicotine use, suffering withdrawal symptoms if usage is halted. Human studies have reported cognitive-enhancing effects, such as improvement of fine motor functions, attention, working memory, and episodic memory, in long-term use of nicotine after withdrawal alleviation. These positive reinforcing effects may further encourage the maintenance and continuation of nicotine use due to unpleasant side effects if cessation occurs. Cognitive side effects may include difficulty concentrating, impaired attention, and impaired working memory [1,13].

The mesolimbic dopamine (DA) pathway, important for reward and addiction behavior, as well as the nicotinic acetylcholine receptors (nAChRs) are implicated to be involved in the cognitive and pleasurable effects of nicotine [1,2,4,21,22]. The mesolimbic DA pathway connects the ventral tegmental area (VTA) in the midbrain to the ventral striatum in the forebrain. The main source of reinforcement signaling is the DAergic neurons located in the VTA and the substantia nigra pars compacta (SNc). When the mesolimbic DA pathway is in a resting state, DAergic neurons fire in a tonic pattern, steady and with low frequency. However, when a stimulus is introduced, the response becomes a phasic firing pattern, encouraging goal-motivated behaviors. Substances that are often abuses, such as nicotine, act as a stimulus resulting in this increase in DA firing. The prolonged DA release has previously been hypothesized to be a motivational influence associated with continued drug use and involves several brain regions (prefrontal cortex, hippocampus, amygdala, and dorsal and ventral striatum). The altered DAergic signaling in these brain regions lead to behavioral and cognitive effects seen with chronic nicotine usage, including increased anxiety-like behavior attentional deficits, depression, and addiction [21,23].

**Figure 1:**
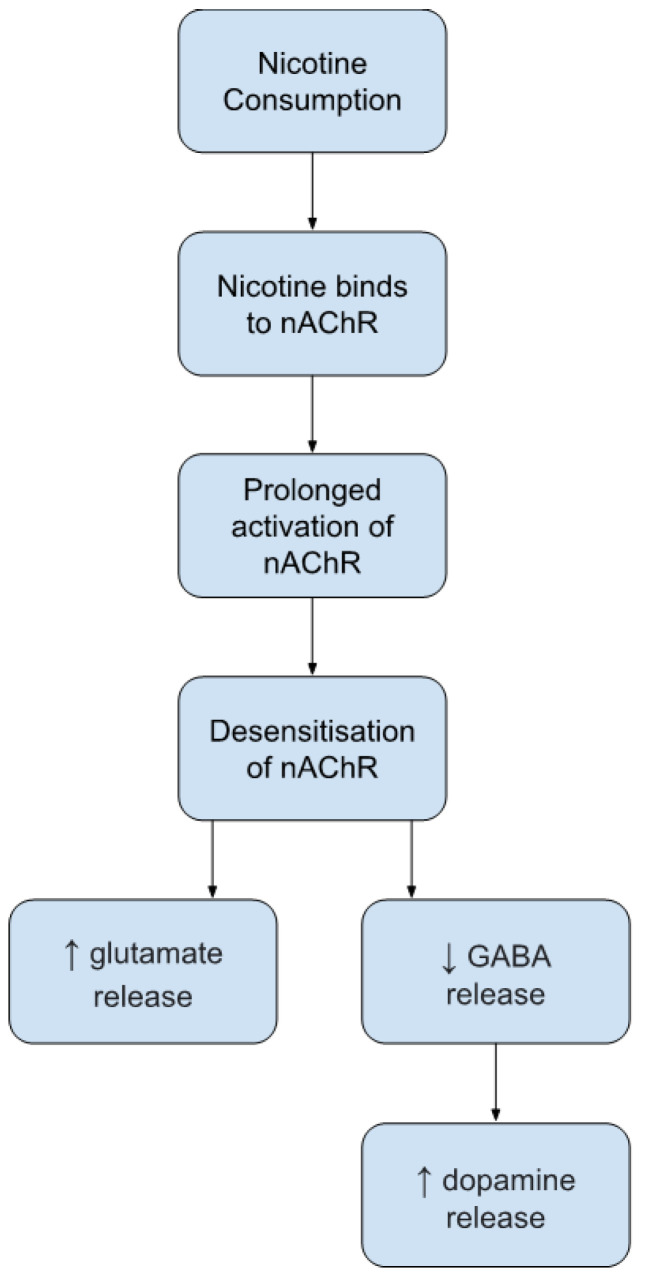
Nicotine mechanism of action.

In addition to dopamine, nAChRs are ligand-gated ion channels consisting of nine α and three β subunits arranged around a central permeable pore. These receptors are the primary site of drug action for nicotine. Nicotinic receptors are located on GABAergic interneurons and DAergic neurons in the VTA along excitatory and inhibitory input axons and/or terminals [1,21,24]. When acetylcholine binds to nAChRs, the enzyme acetylcholinesterase inactivates the neurotransmitter within milliseconds. However, when nicotine binds to these receptors, it is not a substrate for acetylcholinesterase, and therefore, prolonged activation of the nAChR occurs. This prolonged activation can lead to desensitization of the nAChRs, which may result in a greater glutamate release compared to GABA after prolonged nicotine exposure. This GABA deficiency may lead to an enhanced DA release in the nucleus accumbens, a potentially important driving mechanism for continued nicotine usage [1]. Figure 1 provides a summary of these mechanisms of action. 

The maladaptive behaviors associated with obesity and nicotine share commonalities on both a behavioral and neurobiological level; however, the extent of their overlap is less explored and understood. The relationship between smoking and obesity is not a linear one. There are many factors, such as the length of time using nicotine and the amount of nicotine consumed, that must be taken into consideration. People who are current smokers are often observed as leaner when compared to those who have never smoked before. However, long-term smokers are more likely to be considered overweight. Additionally, heavy smokers are more likely to have obesity compared to moderate or light smokers [2,25,26]. The relationship between obesity and nicotine use can be explored amongst all age groups.

## 2. Obesity

### 2.1. Epidemiology of Obesity and Clinical Significance

Environmental factors that may contribute to obesity include decreased physical activity and increased energy consumption. These relationships have been widely studied [2,5,15,27,28,29,30]. Socioeconomic status and parental education have also appeared to be potential environmental factors. Low socioeconomic status and parental education are associated with numerous consequences that are relevant to obesity risks. Those dealing with financial hardships have less access to healthy lifestyle choices and indirectly increase consumption of unhealthy and calorically dense food [15,31,32,33,34]. In the past 30 years specifically, obesity has become a public health concern among individuals of all economic statuses, ages, and lifestyles [15,16,17,27,28,31,32,33].

There are a number of co-morbidities associated with obesity. These can include sleep disturbance, respiratory difficulties, joint and mobility issues, psychological distress, type 2 diabetes mellitus, hypertension, dyslipidemia, cardiovascular diseases, and certain types of cancer [15,28]. Along with the many physical effects resulting from obesity, there are also social implications that follow. Individuals with obesity often face prejudice and are viewed in a negative light. Associations between exposure to tobacco smoke during prenatal and early postnatal life have been shown to increase the risk of obesity in young children. Evidence also suggests that once obesity is established in childhood, it is difficult to reverse and may continue into and through adulthood [16,17].

### 2.2. Obesity and Food Addiction: Mechanisms of Action

Food addiction (FA) has no current medical diagnosis, but the behavioral deficits and dysregulation of brain reward systems that occur are similar to the effects seen with common substances of abuse [2]. High-fat and high-sugar food consumption is commonly associated with an increased risk of obesity [29,35]. Like the neurological mechanisms behind nicotine addiction, the mesolimbic reward pathway and DAergic neurons are believed to be connected to behaviors associated with obesity [36]. Several hypothalamic neuropeptides play a role in FA, regulating processes such as feeding and energy metabolism, including leptin [30,37,38], insulin [22,38], ghrelin [39,40], orexin [41,42], cholecystokinin (CCK) [43,44], peptide YY (PYY) [45,46], and neuropeptide Y [45,47]. The mechanisms of action for these peptides connect the to the dopaminergic system as well [22,33,37,38].

High-fat food appears to activate the hippocampus and caudate, which are, in part, responsible for associative learning. High-sugar foods, on the other hand, appear to activate areas of the DA pathway, indicating more effective recruitment of reward regions [35]. These mechanisms have previously been explored in animal models. In one study, when rats were on a diet with intermittent access to sugar, a longer-lasting increase in acetylcholine levels and DA release in response to sugar consumption continuously increased compared to the baseline over 21 days [29]. It is unclear, however, if the involvement of these reward-related pathways is a response to weight gain or poor diet or if they precede the development of obesity. Similarly seen in humans, rodent models show that individuals can either be obesity-resistant (OR) or obesity-prone (OP), each affecting DA circuitry differently. In these rodent models, extracellular DA levels in the nucleus accumbens (NAc) shell were recorded as lower in OP rodents compared to OR rodents. When given a high-fat meal, increased DA release occurred in both rats, but levels of DA in the NAc in OP rats remained lower over time [36]. Generally, in the NAc, DA release has been associated with reinforcing effects of food. In the hypothalamus, however, DA release is associated with the duration of meal consumption and is required for initiation of a meal along with the quantity and length [30].

DA receptor D2 (D2R) expression has also been considered an important part of the mechanism for obesity although discrepancies in the literature exist. The involvement of D2R does not appear to be directly involved in weight status but rather the eating behaviors leading to weight gain [2,48,49,50,51]. Lower D2R expression in individuals with obesity would cause a lessened sensitivity to reward stimuli, causing an increased vulnerability to food intake as a means to temporarily compensate for the deficit [30,52]. An association between low D2Rs in the striatum and activity in the dorsolateral prefrontal cortex, medial orbitofrontal cortex, and cingulate gyrus has previously been seen. These brain regions are associated with inhibitory control, salience attribution, and emotional reactivity, with disruption giving rise to impulsive and compulsive behaviors, which could be associated with a higher risk of overeating. A significant association between striatal D2Rs and metabolism in somatosensory cortices also appears to exist. These regions are responsible for processing palatability and could underlie one of the mechanisms through which DA regulates the reinforcing properties of food [49]. Food cues have been seen to increase striatal extracellular DA and increase metabolism in the orbitofrontal cortex as supporting evidence for involvement of DA in non-hedonic motivational properties of food [48,53]. Another study reported that striatal D2R availability was reduced in subjects with obesity, similar to drug-addicted individuals. This and other studies provide support that this reduction may predispose subjects with obesity to seek food as temporary compensation for under-stimulated reward circuits, similar to what is seen in drug abuse [30,48,50,54].

The observation of reduced availability of D2R compared to body mass index (BMI) in individuals with obesity may be potentially leading to prolonged disordered eating to balance the reduced stimulation of reward and incentive circuits [54]. Low D2R density has been associated with risk of relapse of substance abuse, including glucose craving and nicotine sensitization [55]. Findings in the Davis et al. [50] study provide further support of this association. Using the D2R agonist, bromocriptine, this rat model provides further support of unique leptin-DA interactions and the hypothesis that there is hyposensitivity of the DA system in obesity. Furthermore, research on Zucker obese rats has shown a robust difference compared to control rats in DA activity, including the role of D2R levels in the brain that are modulated by food. Rats who were food-restricted had significant and greater D2R levels compared to those who were unrestricted [50,51,56]. Moreover, in vivo imaging research has shown that D2R binding availability in the brain can be used to predict future body weight levels and cocaine preference (drug abuse) [57]. This supports D2R involvement in eating behaviors. 

Another study saw similar results with a high-fat diet in rats. On a chronic high-fat diet, rats became obese with reduced D1R-like binding in the ventrolateral striatum and NAc core, reduced D2R-like binding in all areas of the striatum, and reduced dopamine transporter (DAT) binding within all areas of the striatum [58]. Production of D2Rs has also been observed to decrease in striatum of mice fed a high-fat diet via an increase in DNA methylation of the D2R promoter region [59]. Using the D2R agonist bromocriptine, a rat model observed an increased motivation for procuring high-fat food. These findings support the susceptibility to obesity relating to differential sensitivity to D2R stimulation [52].

## 3. Genetic Factors of Nicotine Use and Obesity Risk

Translational noninvasive neuroimaging studies have yielded a plethora of evidence that drug addiction and obesity share striking similarities in functional impairment in discrete brain regions and neurotransmitter circuits (for review, see [30,60,61,62,63,64]. Reward-deficiency syndrome (RDS) describes deficit conditions in mesocorticolimbic DA function leading to abnormal craving behaviors. It was proposed that a disturbance along the brain reward cascade caused by both genetic and epigenetic factors can result in the addictive behaviors defining RDS [65,66,67,68,69,70]. While the polygenic inheritance of obesity is accepted, one of the most prominent of these genetic risk alleles for RDS is within the DA receptor gene (DRD2), and the Taq1 A1 allele. The presence of this allele results in attenuated DA receptor levels. Therefore, DA function, a key player in both substance abuse and obesity, is reduced [9,10]. RDS manifests as an individual’s neurochemical deficiency at perceiving reward, including DA function deficiency and alterations in reward pathways, resulting in the seeking behavior of alternative ways to promote DA release, such as substance abuse and overeating [65,71,72,73,74,75,76,77,78,79,80,81,82,83,84,85,86,87,88,89], binge eating, etc. [90,91,92,93,94,95,96,97,98,99,100,101,102,103,104]. Individuals, for example, carrying the DRD2 Taq1 A1 have a predisposition for reward-seeking behaviors due to DA function deficiency and alterations in reward pathways [65,105,106,107,108,109,110,111]. This concept supports that DR2 is not directly or exclusively involved in an individual having obesity but rather the behaviors and eating mechanisms potentially leading to obesity. Previous research has demonstrated the DRD2 Taq1 A1 allele is present in 67% of overweight and obese subjects, accounting for 45.9% of the variance of all cases with more than 34% body fat [71]. These results were also supported by other studies showing that parent–child concordance of DRD2 Taq1 A1 allele predicted similarity of parent–child weight loss [112] and that there was a significant correlation of Taq1 A1 with percent body fat in obese subjects [71]. Finally, the DRD2 Taq1 A1 allele significantly correlated with other known obesity-related gene variants, such as the OB1875 allele, and this correlation was even stronger in women [113]. Preclinical studies have tested this hypothesis by genetically upregulating D2R levels in the brain and showed an attenuation in substance abuse [30,114,115,116,117]. When examining D2R levels in non-drug abusing individuals given the stimulant drug methylphenidate, those who experienced a pleasant feeling had lower levels, while those who reported an unpleasant feeling expressed higher levels. This information, although not sufficient alone, provides support that D2R levels may also contribute to an individual’s vulnerability towards addiction [30,71,118]. 

The consumption of nicotine stimulates the production and utilization of DA in the brain [119,120,121], which would further encourage nicotine use in individuals with hypodopaminergic signaling. An individual with hypodopaminergic signaling (or RDS) may be more vulnerable to substance use, such as smoking [122,123,124,125,126,127], even during pregnancy [128,129,130,131,132,133,134,135,136,137,138,139], leading to potential obesity risks later on in offspring [65,66,91,103,113,140,141,142,143,144,145,146,147,148]. If this genetic risk is passed down as well, offspring may also then experience abnormal craving behaviors associated with both obesity and substance abuse. An animal study observed hyperphagic behavior and preference for a high-sucrose diet in female rats exposed to nicotine during lactation, coinciding with an increase in protein content of DRD2 in the NAc [134]. There is also evidence that smoking significantly increases DRD2-gene methylation, which contributes to both substance and non-substance addictions [79,149]. These results continue to further support the role of alterations of DRD2 as part of the mechanism leading to obesity.

Previous research has demonstrated various genetic risk alleles associated with obesity and substance abuse (dopamine D1 Receptor (DRD1): rs4532—risk allele G, dopamine D2 receptor (DRD2): rs1800497—risk allele A1, dopamine D3 receptor (DRD3): rs6280—risk allele C (Ser9Gly), dopamine D4 receptor (DRD4): rs1800955—risk allele C (48bp repeat VNTR), dopamine transporter receptor (DAT1): SLC6A3 3′-UTR—risk allele A9 (40bp repeat VNTR), Catechol-O-Methyltransferase (COMT): rs4680—risk allele G (Val158Met), µ-opioid receptor (OPRM1): rs1799971—risk allele G (A118G), γ-aminobutyric acid (GABA) A receptor, β-3 subunit (GABRB3): CA repeat—risk allele 181, monoamine oxidase A (MAO-A): 3′ 30bp VNTR—risk allele 4R DNRP, serotonin transporter receptor (5HTT)-linked promoter region (5HTTLPR) in SLC6A4: rs25531—risk allele S′). These alleles were proposed for a GARS panel in case-control studies for numerous substance abuse disorders, including nicotine (see Table 1). It is important to be able to examine their existence prior to the consequence. The Genetic Addiction Risk Severity (GARS) was developed to test and predict an individual’s vulnerability to pain, addiction, and compulsive behaviors defining RDS [67,105,106,150,151,152,153,154,155,156,157,158,159,160,161,162]. It examines a panel of 11 reward gene risk variants with established polymorphisms reflecting the brain reward cascade and predicting the severity of potential drug and alcohol dependency. The identification of genetic risks for RDS is essential in providing aid for treatment and prevention [67]. For prenatally nicotine-exposed individuals as well as others with increased obesity and substance abuse risk, identification of these genetic risk alleles and polymorphisms may allow for early intervention.

### Prenatal Nicotine Exposure and Genetic Risk Factors for Obesity

One approach for investigating obesity in children involving prenatal nicotine exposure examines genetic risk factors. Substance use disorder (alcohol, drugs, and food binge-eating disorder [66]) is a result of gene and environment interaction. The shift from the initial use of a substance to a progressive pattern of use despite catastrophic consequences is influenced by intrinsic and extrinsic factors as well as the nature of the addictive agent [172]. Often, theories look at obesity risks in offspring as a direct result of prenatal behaviors of the mother, considered an environmental influence. Genetic risk factors, however, are also critical and contribute to influence the behavior of the offspring in a more indirect manner, especially in the transition to more problematic usage. For example, even in the absence of maternal smoking during pregnancy, there may be a genetic risk inherited by offspring leading to increased vulnerability for obesity or smoking, similar to that following prenatal exposure. Specific genetic risk alleles (i.e., DRD2 Taq1 A1) that are carried and passed on by individuals contribute to these consequential behaviors [54,65,67,172].

Examining specific gene clusters, the CHRNA5-CHRNA3-CHRNB4 gene cluster on chromosome 15q25 has shown the strongest evidence of association for smoking behaviors. It is this gene cluster that encodes for nicotinic acetylcholine receptor subunits alpha five, three, and beta four, which are a part of the primary site of drug action for nicotine [24,126,173]. Single-nucleotide polymorphisms in the region are repeatedly associated with smoking behaviors. For example, the CHRNA3-rs1051730 asparagine allele has been associated with nicotine dependence and smoking quantity [126,174,175]. One specific study examined maternal smoking effects on offspring in relation to these gene clusters. Results showed a significant correlation found for the CHRNA3-rs1051730 asparagine allele. Offspring of mothers smoking heavily during pregnancy were more likely carrying this allele compared to offspring of non-smoking mothers [126,172,174].

MAO genes have also been shown important smoking behavior with considerable individual and sex-related differences. For example, the MAOB G variant has been seen to be more prevalent in female smokers than nonsmokers, while the MAOA 1460 T-variant is the opposite [176].

## 4. Other Hypothesized Mechanisms of Nicotine and Obesity

### 4.1. Prenatal Nicotine Exposure and Obesity

In the developing brain, some of these nAChRs begin to be expressed in the pons and midbrain in the early first trimester as soon as 5 weeks after conception. With the early development of these receptors, the deleterious effects of prenatal nicotine exposure, including alterations in expression and function of nAChRs within the central nervous system (CNS), are not surprising to be observed. Nicotine’s premature stimulation in cholinergic signaling and desensitization of nAChRs consequences is therefore expressed in a developing brain already undergoing numerous dramatic changes [4].

**Figure 2:**
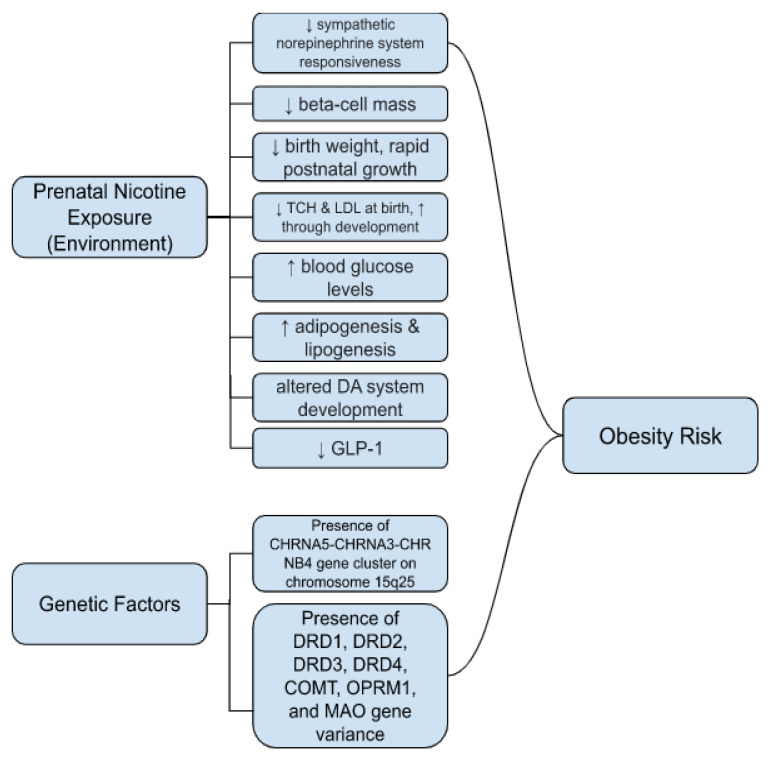
Summary of Physiological Effects in Offspring Prenatally Exposed to Nicotine through Maternal Smoking.

Previous research has shown that prenatal nicotine exposure does have an effect on obesity risks, but the relationship is complicated. Children who were prenatally exposed to tobacco smoke have a 37% and 55% increased risk of being overweight or having obesity, respectively, compared to children of nonsmokers [17]. Figure 2 provides a summary of physiological effects that have been observed in offspring who experienced prenatal nicotine exposure. It has been seen that children of smokers are more frequently born with low birth weight compared to those of nonsmokers. However, due to possible mechanisms such as catch-up growth, these children are seen to be at a greater risk for over-weight status later in development [8,16,20,38,177]. A further explanation of catch-up growth and the mechanisms behind the theory is referenced in Section 4.3. In general, children with a high birth weight also have an increased risk of later obesity; however, the low birth weight often associated with prenatal nicotine exposure can also lead to a predisposition for obesity [17]. Other possible mechanisms have also been considered as potential links between parental smoking and childhood obesity risks. Besides catch-up growth, the thrifty phenotype theory has been considered as well as a neurotransmitter or endocrine hormone imbalances. These theories examine how environmental factors, such as nicotine exposure, lead to adaptations in development and endocrine systems. Further explanation of these theories is explained in Section 4.5 and Section 4.6.

The relationship is also complex due to the possibility a dose-dependent relationship between nicotine and obesity may exist. 

In a study including both maternal and paternal smoking, maternal smoking during pregnancy was associated with increased risk for overweight and obesity in adulthood [178]. There were dose-dependent associations with increased obesity risk related to higher number of cigarettes smoked per day during pregnancy. Furthermore, the association was strongest among daughters of women who smoked 25 or more cigarettes per day during pregnancy. The significant effects observed for maternal smoking in this particular study, when compared to paternal smoking, suggest a direct intrauterine effect on the child’s body size. Different mechanisms suggested for the influence on body size were through the programming of the fetal hypothalamic–pituitary–adrenal axis or alteration in pancreatic function and appetite control [178,179,180,181]. This study provided important insight into the association of both maternal and paternal smoking during pregnancy and the resulting effects on obesity. It has been seen that significant deficits in offspring of male mice exposed to nicotine bred with drug-naïve mice occurred in attention, brain monoamine content, and dopamine receptor mRNA expression, so it is possible that paternal smoking may have a role as well [137]. In the Harris et al. (2013) study, smoking behavior was measured by the number of cigarettes smoked per day and the effects on birth weight, childhood body size, and BMI throughout adulthood. This specific study is especially important to the topic, as there have been few studies examining the association with body size later into adult life, including both maternal and paternal smoking, examining potential dose-response and sex-specific associations. Results indicated that maternal smoking during pregnancy was independently associated in a dose-response manner with an increase in the risk of body size from childhood through adolescence and into adulthood in the daughter [178]. Confounding variables on both the maternal and paternal side exist and are of importance in the relationship between smoking and childhood obesity. Some of these variables include parental socioeconomic position, education, lifestyle factors, and dietary habits [16,17].

### 4.2. The Barker Hypothesis

In the early 1990s and going forward, David Barker and colleagues explored the in-verse relationship between birthweight and risk of adult diseases in more depth. Adult coronary heart disease, stroke, hypertension, and type 2 diabetes were specifically observed. The findings of this study were attributed to a period of developmental plasticity [182]. During this growth period, the fetus responds to its environment with adaptive changes in organ structure and metabolism based on fetal nutrient delivery. These changes, while necessary for survival at the time, may become maladaptive and lead to increased risk of disease later in life. This became known as the developmental origins hypothesis, commonly referred to as the Barker hypothesis [19,182]. This hypothesis is important in providing support for disease and obesity in offspring, potentially because of prenatal nicotine exposure (see Section 5 for detailed data supporting this hypothesis from preclinical and clinical models). Environmental chemical exposure, such as nicotine from maternal smoking, may interfere with intrauterine nutrient supply, among many adverse effects [4,9,10,14,15,16,17,19]. Because of the limited nutrient supply the fetus experiences, it must adopt what is known as a “thrifty” phenotype to increase energy efficiency. This phenotype can include decreased lipid metabolism, lower muscle mass, decreased insulin secretion, and decreased nephron number, for example, in turn elevating the risk of metabolic syndrome and related diseases [19].

### 4.3. Catch-Up Growth Theory

One of the possible mechanisms for postnatal childhood and adolescent obesity is the theory of catch-up growth. Smoking during pregnancy is well-documented to result in lower birth weight. This can be caused by greater vascular resistance, noted as chronic and acute enhanced blood flow velocity in different fetal vascular beds as well as enhanced blood flow velocity in uterine arteries. The combination of increased resistance and reduced flow restricts fetal growth in smoking mothers. Nitric oxide (NO), a vasodilator, has also been considered a mechanism for the intrauterine growth restriction seen. Deficiency in NO production plays a role in the reduced blood flow due to smoking [20]. With intrauterine growth restriction occurring, offspring are at greater risk of low birth weight. The theory of catch-up growth refers to the accelerated growth of a low-birth-weight child through infancy in order to compensate. One study amongst many recorded a clear early postnatal catch-up growth leading to increased risk of central and peripheral obesity in childhood and potential risk for disease in adulthood. In this specific study, children who were seen to have catch-up growth between zero and two years old developed more central fat distribution at five years compared to other children [177]. Limited studies have been conducted on the influence of low birthweight and catch-up growth in early childhood; therefore, more research would need to occur to solidify the theory.

### 4.4. Thrifty Phenotype Theory

The thrifty phenotype theory centers on the hypothesis that “early-life metabolic adaptations promote survival, with the developing organism responding to cues of environmental quality by selecting an appropriate trajectory of growth” [183]. Consistent with the Barker hypothesis, the work of Hales and Barker proposed that early malnutrition in fetal development invoked physiological compensation. This occurred as a means to promote early survival but at the cost of later health issues. The poor fetal environment, in the case of prenatal nicotine exposure, is identified as having less than optimal nutrition [18,183,184,185]. A cascade of negative changes may follow as a result of having to adapt to the poor environment. These could include alterations of hypothalamic functioning and lowered insulin secretion and resistance [18,19,183]. Consistent with this concept is the idea that a child born with poor fetal growth will experience rapid growth postnatally, which potentially leads to obesity. Maternal smoking replaces malnutrition as the stressor inducing the adaptations. This theory is the incorporation of catch-up growth, with further exploration into the causes of rapid postnatal weight gain. An individual exposed prenatally to stressors must adapt to their current environment as a means of survival. For individuals of low birth weight due to prenatal nicotine exposure, the individual experiences rapid weight gain for survival in the immediate time frame. This occurs, however, at the cost of obesity later in development.

### 4.5. Neurotransmitter or Endocrine Imbalances Theory

Central and peripheral sympathetic norepinephrine (NE) systems are seen to control appetite regulation and adipose metabolism. Fetal nicotinic overload due to maternal smoking may cause blunting of these systems, contributing to overeating and increased weight gain. Due to the overload, normal feedback mechanisms responsible for curbing appetite and decreasing lipogenic responses are blunted as a possible potentiation for obesity [5]. During prenatal development, the sympathetic nervous system connections and reactivity are programmed. Damage during this development has been seen to cause long-lasting impairment of sympathetic response lasting into adulthood. Early toxicant exposure, such as to nicotine, during this sensitive time period may result in a potentially permanent alteration of the sympathetic nervous system [186]. Nicotinic cholinergic mechanisms have been seen to play a major role in the programming of central and sympathetic nervous system development. Nicotine acts as a nicotinic cholinergic agonist, impairing this development and therefore affecting NE systems as a result. These systems are critically involved in the control of appetite and adipose metabolism, so when blunted and less responsive, increased obesity risk in the offspring can occur. The prenatal nicotinic overload impairing responsiveness on the peripheral and central noradrenergic system’s under-activity leads to increased appetite and decreased mobilization of fat from adipose tissue [5]. The cascading effects from the initial prenatal nicotine exposure provide the possible mechanism of neurotransmitter or endocrine imbalances for obesity in offspring.

Glucogan-like peptide 1 (GLP-1) is a gut hormone involved in potentiating insulin secretion and inhibiting glucagon secretion, reducing appetite, and slowing gastric emptying [187]. GLP-1 receptors are also expressed in numerous brain regions associated with reward–addiction pathways, making GLP-1 a potential candidate for involvement in the relationship between nicotine and obesity. A study previously observed that with increased simple sugar intake, GLP-1 signaling for satiation was reduced, potentially leading to overeating and weight gain. These abnormal activities were not observed in lean individuals, as seen in individuals with obesity specifically in the NaC [188]. Similar lack of satiating behavior has been observed involving GLP-1 and nicotine in preclinical models [189]. GLP-1 has been suggested to mediate nicotine intake similar to that of meal patterning, with activation inducing satiating sensors to indicate overconsumption prior to the appearance of adverse effects [190]. For preclinically exposed individuals with the potential to develop obesity, reduced GLP-1 signaling could be associated with lack of these sensors, more greatly increasing this risk for weight gain. 

### 4.6. Adipogenesis, Lipogenesis, and Glucose Metabolism

Obesity can be described as an accumulation of excessive fat tissue, more specifically an excessive secretion of the endocrine organ, white adipose tissue (WAT). Adipogenesis is the formation of adipocytes, which are cells responsible for fat storage. Literature has previously suggested prenatal nicotine exposure altering early adipogenesis as a mechanism for increased obesity susceptibility. A study aimed to investigate perinatal nicotine exposure effects on early adipogenesis and then consequently programmed body weight, body adiposity, and metabolic alterations in adult male Wistar rats [11]. Results showed that prenatal nicotine exposure resulted in higher body weight and fat content at the time of weaning. This mainly appears to be due to hyperplasia of adipocytes that are associated with increased gene expression of those involved in adipocyte differentiation and lipid synthesis. The nicotine-exposed adult male offspring were observed to have increased levels of adipogenic and lipogenic genes, glucose transporter 4, and leptin mRNA [11]. These are associated with increased adipogenesis and lipogenesis. It is important to note that food intake between nicotine and control groups was unchanged; therefore, the recorded differences in body weight and body adiposity were not a result of excessive food consumption. Although the developmental timing of adipose tissue formation differs in rats compared to humans, the findings of this study provide strong evidence of adult body weight being programmed in early life and therefore prenatal nicotine exposure contributing to obesity and other metabolic disorders through adulthood [11].

In another animal model, brown adipose tissue (BAT), which is associated with obesity and energy metabolism, was examined in rats. This study looked at the effects of nicotine administration in pregnant rats and the resulting consequences in male offspring. Results showed a decreased brown-like phenotype in BAT of the male offspring in adulthood from downregulation of the AMPK-SIRT1-PGC-1α pathway, believed to be a possible mechanism of nicotine-induced obesity [191].

Obesity is also associated with glucose intolerance and impaired glucose metabolism [6,7]. Using glucose tolerance tests, multiple animal studies observed a reduction in glucose use and delayed glucose clearance in male rat offspring postnatal life at 26 weeks of age following prenatal nicotine exposure [11,192]. Results showed nicotine exposure at concentrations representative of human exposure during pregnancy and lactation was consistent with disturbed glucose metabolism possibly leading to type 2 diabetes (T2D) [192]. In another study examining glucose concentration, subjects with obesity after a 5-year follow-up showed the greatest deterioration of glucose tolerance [193]. These individuals were more likely to develop T2D. The evidence presented in these studies supports glucose as a potential mechanism for childhood obesity following prenatal nicotine exposure.

### 4.7. Prenatal Nicotine Exposure and Metabolic Disease

Both chronic nicotine exposure and obesity result in numerous maladaptive side effects and potential co-morbidities of psychological and physiological disorders. Metabolic syndrome refers to obesity and its consequential related complications, including impaired glucose metabolism, elevated blood pressure, and dyslipidemia. For example, studies have previously seen an association with elevated systolic or diastolic blood pressure associated with maternal smoking before birth through childhood [8,194]. One study further explored this association, observing a small long-term effect on adolescent and young adults who were exposed to maternal smoking. Whether this association remains through adulthood remains uncertain, but a significant increase in both systolic and diastolic blood pressure in young men whose mothers smoked throughout pregnancy was observed [195]. Children born with prenatal nicotine exposure via maternal smoking are often born with low birth weight. This lower birth weight due to potential fetal malnutrition may result in an imbalance in metabolism resulting in an array of potential diseases, including metabolic syndrome, later in life [6,7,19,25,183].

#### 4.7.1. Gestational Diabetes Mellitus

Gestational diabetes mellitus (GDM) is a condition seen in pregnant women occur-ring when a buildup of glucose in the blood prevents insulin from being used effectively, affecting the fetus. GDM is observed to lead to an increased risk of metabolic syndrome and type 2 diabetes in both the mother and offspring [183,196,197,198,199]. Secondary data analysis of the Pregnancy Risk Assessment Monitoring System 2009–1015 observed prenatal smoking was associated with a higher likelihood of self-reported GDM after adjusting for known risk factors. In this study, inadequate gestational weight gain was associated with an increase in odds of GDM [200] in women who continued to smoke throughout pregnancy. The physiological mechanisms underlying the higher risk of GDM in pregnant women may coincide with the mechanisms underlying higher diabetes risk in smokers: insulin resistance and impaired glucose hemostasis. In both animal and human studies, nicotine has been found to reduce the release of insulin through direct activation of nicotinic receptors on pancreatic islet cells [201,202,203].

#### 4.7.2. Type 1 Diabetes

The relationship between both type 1 (T1D) and type 2 diabetes (T2D) in offspring exposed to nicotine prenatally is one that has not been explored greatly. T1D is one of the most common autoimmune diseases in children and young adults and is caused by immune-mediated beta-cell destruction [202]. A review on maternal exposure to smoking during pregnancy and T1D in offspring found numerous studies that did not support an association between maternal or paternal smoking during pregnancy and T1D in their offspring [204,205,206,207,208,209,210]. However, in an animal study using mice, decreased body weight and elevated blood glucose levels indicated that prenatal nicotine exposure caused persistently impaired glucose homeostasis, which are metabolic changes consistent with T1D characteristics [202]. Although further research is needed to explore the relationship between prenatal nicotine exposure and T1D, studies such as these suggest a potential relationship does exist.

#### 4.7.3. Type 2 Diabetes

T2D is often categorized as a part of the metabolic syndrome associated with obesity. It is characterized as a gradual decline in pancreatic insulin secretion and worsening hyperglycemia [211]. An animal study using rats reported that when exposed to nicotine during gestation and lactation, apoptosis of insulin-secreting cells, increased postnatal weight, and abnormal glucose tolerance occurred [192]. This fetal and neonatal exposure to nicotine resulted in a syndrome in rats with characteristics similar to human T2D. Not only are fetal tissues at risk for chemical damage but beta-cell proliferation and beta-cell apoptosis as well. Beta-cell proliferation is important in the early postnatal period for an increase in endocrine cells. A study found beta-cell mass decreased by about 25% after nicotine exposure both in utero or fetal and neonatal but recovered in animals not exposed during lactation. These findings suggested nicotine can destroy cells responsible for beta-cell regeneration if present pre- and postnatally [211,212]. Poor fetal and early postnatal into infancy nutrition has been observed to be detrimental to the development and function of beta cells, with such defects leading to a predisposition of T2D [184]. Prenatal nicotine exposure is associated with poor intrauterine nutrient supply.

Nicotine is also seen to inhibit the release of insulin from isolated islets and decrease beta-cell mass. This decrease allowed the apoptosis regulator gene, Bax, to attach to the mitochondria, opening the permeability transition pore to release cytochrome c into the cytosol. Cytochrome c then formed an apoptosome and activated caspase 3, a key protease responsible for apoptotic changes and cell death [211,212]. The nicotine-induced damage to beta cells and the coinciding development of T2D can lead to cascading side effects. Deterioration of the vascular endothelium, increased pulse pressure and pulse wave velocity, and lack of vasodilating action of nitric oxide allowing deterioration of tissue perfusion are examples. Side effects such as these resulting in aortic stiffness may also increase the risk of cardiovascular disease, a major concern for T2D [192,211,212,213].

#### 4.7.4. Cholesterol

A major pathological characteristic of obesity is the excess accumulation of adipose tissue. Adipocytes are the predominant cell type in this tissue, used to store excess energy and secrete hormones. This type of cell is also key in maintaining cellular cholesterol homeostasis through communication between the adipose tissue-free cholesterol depot and the blood cholesterol pool. Previous literature has seen a strong correlation in adipocyte dysfunction in both obesity and cholesterol imbalance. In individuals with obesity, adipocytes and therefore adipose tissue are seen to contain considerable levels of cholesterol [214].

Cholesterol is also important for fetal development, with a lack resulting in severe birth defects. Exposure to nicotine during pregnancy has been seen to induce abnormal cholesterol levels in the fetus [215,216]. Effects of cholesterol following prenatal nicotine exposure have been observed in both preclinical and clinical studies. An animal study using Wistar rats specifically examined the placental mechanism of prenatal nicotine-exposure-induced abnormal blood cholesterol levels in the fetal rats. Results showed prenatal nicotine exposure resulted in decreased levels of blood total cholesterol (TCH) and low-density lipoprotein-cholesterol (LDL-C) in female fetal rats [215].

A 27-year follow-up study examined the association of maternal smoking in pregnancy with the development of cholesterol levels from childhood to adulthood. Believed to be the first of its kind, the study’s results showed that prenatal nicotine exposure through smoking during pregnancy was associated with an increased annual rise in total cholesterol levels from childhood through adulthood [217]. Similar to the animal model, TCH levels were lower in early childhood, increasing onward through adolescence. Most importantly for this review, it was demonstrated in a stratified analysis that the effect of maternal smoking during pregnancy on total cholesterol development was restricted to offspring who were moderately overweight, defined as a body mass index standard deviation greater than one. This provides an indication that offspring with prenatal nicotine exposure and who develop overweight themselves are at higher risk of developing higher cholesterol levels [217].

#### 4.7.5. Liver Disease

Obesity has been associated with an increased risk in several liver diseases, including non-alcoholic fatty liver disease (NAFLD). NAFLD is among the most common liver diseases and is the most common cause of chronic liver disease. NAFLD reported a prevalence as high as 80% in obese patients in comparison to 16% in those reporting a normal BMI and without metabolic risk factors [218,219,220]. The most defining feature of NAFLD is steatosis. This condition is characterized by an increase in intrahepatic triglyceride (IHTG) content. This can occur with or without inflammation and fibrosis as well. NAFLD can occur through two stages of establishment. The first is associated with metabolic change, characterized by an increase in free fatty acids and de novo lipogenesis, leading to steatosis. The second is associated with reactive oxygen species’ deleterious effect on cell structure, insulin resistance, and the release of proinflammatory cytokines. The development of NAFLD is a major health concern, as it can progress to severe liver disease and is associated with other severe cardiometabolic abnormalities, such as T2D mellitus and metabolic syndrome [221,222]. Previously, it has been demonstrated that smoking can contribute to the development of this liver disease, as nicotine is mainly metabolized here.

In an animal study using male Wistar rats at 10 weeks of age and with chronic nicotine exposure, an impairment of lipid metabolism and micro steatosis was observed. However, the mechanism of nicotine toxicity on the rat liver was not fully understood [223]. Another rat model examined long-lasting effects of maternal nicotine exposure upon the oxidative stress markers and antioxidant capacity in plasma and liver, as NAFLD is correlated with a decrease of antioxidant defenses [222]. Results from this study provided evidence that early postnatal nicotine exposure was capable of programming hepatic oxidative damage. This damage was seen to be responsible for the development of micro steatosis later in adult life. The oxidative stress and liver dysfunction detected in this model aid in understanding the increased risk of metabolic and vascular diseases seen in children with prenatal maternal nicotine exposure [222].

The relationship between metabolic dysfunction and NAFLD does appear to exist, but the mechanisms behind it remain unclear. It is not known if NAFLD is a cause or a resulting consequence of this dysfunction [221]. Further research would need to be con-ducted to examine the mechanisms and pathology of this relationship to better under-stand and treat the disease.

## 5. Prenatal Nicotine Exposure and Obesity Risks through Development

### 5.1. Leading Mechanism Hypotheses on Prenatal Nicotine Exposure and Obesity

Maternal smoking has been well-documented in offspring health risks. Of these, obesity typically is not immediately observed but delayed. In contrast, low birth weight is observed because of intrauterine growth restriction, which is recorded in numerous studies. There is a dose-response relationship between maternal smoking during pregnancy and birth weight [8,16,178,224]. For example, there is a 5% reduction in relative birth weight per pack of cigarettes smoked per day, as seen in [224]. Prenatal nicotine exposure is known to induce deleterious effects even prior to birth, such as ectopic pregnancy, placenta previa, placental abruption, stillbirth, and premature rupture of membrane. In addition, prenatal nicotine exposure can lead to an increased risk to be preterm along with low birth weight, and there is already a predisposed increased risk for metabolic syndrome, including obesity as well as cardiovascular issues later in life [6,7,9,10,14,15]. For example, Barker and colleagues (2006) observed an inverse relationship between decreasing birth weight and an increased risk in adult coronary heart disease, stroke, hypertension, and T2D [182]. The birth weight of a baby is dependent on the gestational age of the fetus at delivery as well as the rate of fetal growth, with nicotine affecting both of these factors. The birth weight of prenatally exposed children has been associated with a 150 to 300 g average reduction [6,225]. Harris et al. (2013) recorded that the birthweight of daughters of smokers averaged 3158 g, while that of nonsmokers was 3335 g [178]. Another study saw a mean 36 g reduction in birth weight for infants prenatally exposed via domestic environmental tobacco smoke and a mean reduction of 146 g in those exposed directly from maternal smoking [226]. This low birth weight due to intrauterine growth restriction is what is observed to potentially lead to the catch-up growth theory leading into childhood. The timing of nicotine exposure throughout pregnancy may also be associated with effects at birth. Smoking in the first trimester has been observed to result in a risk for lower birth weight, and it is possible that a cessation in the second and third trimester has no protective effect or could intensify these risks. Further, exposure to tobacco in the last two trimesters was observed to result in restricted growth at birth. However, accelerated conditional weight for length growth that occurred by 2 years of age in a possible dose–response relationship [18,227]. Obesity risks are more easily observed in the accelerated growth in early infancy for compensation. A summary of these effects from birth and into adulthood in both preclinical and clinical models are listed in Table 2 and Table 3, respectively. Prenatal nicotine exposure is also shown to adversely affect the development of the DAergic system in its entirety. In animal models, gestational nicotine (GN) treatment induces complex alterations in the development of DA systems. An animal model using rats examined the effects of GN on sensitivity to natural rewards among male offspring, such as FA, and drug rewards. Although the exact mechanisms were undetermined, this study confirmed GN exposure produced substantial and long-lasting alterations in neural circuitry, underlying both natural and drug reinforcement likely involving the DAergic systems [228,229,230]. The alterations included increased neuronal activation in the nucleus accumbens core and shell, possibly indicating increased activation of D1 receptors and increased basal c-fos mRNA levels in the prefrontal cortex [229]. Another animal study also provided evidence GN alters corticostriatal DA system development. GN treatment decreased striatal DA transporter (DAT) binding in the nucleus accumbens core in females. In human studies, decreased DAT expression is associated with increased striatal reactivity to rewarding stimuli, potentially enhancing susceptibility to addiction [231]. This effect on development is similar to that of the genetic risk. If the mother prenatally exposes the offspring to nicotine, affecting the development of the DAergic system, the individual may now have a greater predisposition for addictive behaviors associated with obesity and substance abuse.

### 5.2. Prenatal Nicotine Exposure Effects at Early Childhood and Adolescence on Obesity Risks

In early childhood, obesity risks due to prenatal nicotine exposure appear to manifest in weight gain and adverse health effects more visibly compared to birth. There have been numerous studies examining offspring of smoking mothers from birth through adolescence to examine the details of these effects. A meta-analysis of 17 studies found that at a mean of 9 years of age, children of smokers had an increased risk of obesity compared to nonsmokers [8]. Another study observed similar results, with maternal smoking at any time during pregnancy being associated with higher total fat mass in offspring at a mean age of 9.9 years [237]. Other studies have seen an increased risk of obesity as early as 4 years of age in children whose mothers smoked during pregnancy [238]. A systematic review on prenatal nicotine exposure and obesity reported from 14 observational studies children of mothers who smoked during pregnancy in comparison to those who do not smoke saw an elevated risk for overweight at ages 3 to 33 years [239]. In a 7-year follow-up study, at 7 years of age, 9.4% of children were overweight with exposure to smoking during pregnancy alone or postnatally as well being associated. This was more prevalent in girls at 10.4% compared to boys at 8.5%, and an increasing number of cigarettes smoked daily appeared to also influence results [16]. Similar results were also seen in animal models. Offspring of rats exposed to chronic nicotine during pregnancy did not see alterations in weight gain during gestation or birth. However, during juvenile and adolescent age, those rats prenatally exposed to nicotine displayed significantly higher weight [5]. Another study using rats showed that prenatally nicotine-exposed offspring, regardless of the timing of exposure, had a significant increase in body weight in female offspring, starting on postnatal day 35 and persisting increase into adulthood at postnatal day 95 [232]. Significant reductions in body weight seen in prenatally exposed mice were no longer seen on postnatal day 10 in one study using a mice model [233]. Although differences in body weight between the prenatally nicotine-treated offspring and control groups did not continue past day 10, it is important to note this information, as a possible period for catch-up growth can occur. 

Shifting into adolescent years, offspring continue to show greater obesity risks as well as metabolic and other observed disorder characteristics. For example, TCH levels observed as lower in childhood increased throughout adolescence in prenatally exposed children, which may then be associated with various cardiovascular health problems [215,217]. It is during childhood and the adolescence period that the rapid weight gain described by catch-up growth theory exists. A significant difference in offspring BMI for 8- to 11-year-olds from two longitudinal family studies, both male and female, when prenatal cigarette exposure occurred was noted. This effect remained significant for the two later stages, 12–15 and 16–18 years, as well [236]. In another study, 13- to 16-year-old children of heavy cigarette smokers had a significantly higher Ponderal index, a measure indicating higher levels of body fat associated with higher scores [240].

### 5.3. Prenatal Nicotine Exposure Effects at Adulthood on Obesity Risks

Exposure to maternal smoking in utero has been associated with as much as a 34 to 41% increased risk of adult obesity [178]. During prenatal development, sympathetic connection and reactivity are programmed. Toxicant exposure with fetal sympathetic damage causes long-lasting damage of sympathetic responses lasting into adulthood. Sympathetic responses are critically involved in the control of adipose metabolism and appetite, which are major factors leading to obesity [5]. It is in adulthood that the Barker hypothesis manifests itself as well. Adult diseases, including coronary artery disease, diabetes mellitus, hypertension, and metabolic syndrome, appear, potentially due to fetal malnutrition as a result of prenatal nicotine exposure [182]. 

These deleterious effects in adulthood can be seen also in animal models. Prenatal nicotine exposure is associated with higher liver oxidative stress and steatosis in adult rat offspring, which has been associated with obesity [222]. Another animal model observed prenatal nicotine exposure affecting early adipogenesis in Wistar rats that lead to programmed body weight, body adiposity, and metabolic alterations in adult life [11]. Believed to be the first of its kind, one study observed fetal nicotine exposure resulted in in-creased adiposity and attenuation of vessel relaxation in adult life in Wistar rats as an underlying mechanism for the increased prevalence of obesity as well as hypertension [234]. 

Examining obesity alone, statistical significance supports an association between maternal smoking during pregnancy leading to obesity in adulthood. Prenatal exposure to tobacco and therefore nicotine results in an increased BMI during childhood, lasting throughout through late adolescence and adulthood [178,236]. Few studies, however, have examined this association with body size in later adulthood; therefore, gaps in the literature may exist. It is also unclear if the association between maternal smoking and adult body mass index is mediated by childhood and adolescent body size [178]. There is robust support for prenatal nicotine exposure leading to obesity and its adverse effects; however, the mechanisms behind it vary, requiring further research for these.

## 6. Conclusions

The relationship between prenatal nicotine exposure and obesity risks exists; however, the exact mechanisms remain complicated. Offspring exposed to nicotine prenatally are observed to have lower birth weight and, as a result, may experience catch-up growth leading to obesity as they enter infancy. There is also a potential genetic risk for prenatally exposed offspring, with the passing on of various genes leading to at-risk behaviors for obesity, such as substance abuse and food addiction. Various environmental factors may also contribute as a mechanism for obesity. A poor fetal environment caused by prenatal nicotine exposure may lead to necessary fetal adaptations to survive, resulting in greater obesity risks, among other negative effects. The increased risk of obesity may also be due to altered reward systems and the effects of both the development and efficacy of the DAergic systems. The effects of prenatal nicotine exposure, specifically on sympathetic responses, last into adulthood and may produce other comorbidities alongside metabolic syndromes, such as diabetes and liver disease. However, future studies should focus on determining if there are long-lasting effects on body size in adults regarding obesity, as gaps in the literature exist. It is important to examine the potential treatment and prevention methods as well as for pregnant women who continue to smoke. 

## Figures and Tables

**Table 1:** Gene polymorphisms under consideration and the literature summary.

Gene/Polymorphism	No. Ref.	Overall Summary
Dopamine D1 Receptor (DRD1): rs4532—risk allele G	[163]	Several studies supported that genetic variation in dopamine receptors D1 may influence genetic predisposition to substance use disorders. A statistically significant association of DRD1 rs4532 polymorphism with nicotine dependence was found among a pooled sample of European American and African American families (2037 participants).
Dopamine D2 Receptor (DRD2): rs1800497—risk allele A1	[83]	The DRD2 rs1800497 was found associated with greater likelihood of nicotine dependence, with individuals carrying one or two copies of the rs1800497 risk allele being 3.3 times more likely in a study (150 smokers vs. 228 controls).
Dopamine D3 Receptor (DRD3): rs6280—risk allele C (Ser9Gly)	[164]	Several case-control studies investigated the association between the DRD3 rs6280 polymorphism with substance use. From a North American study (2037 subjects), the DRD3 rs6280 polymorphism was significantly associated with nicotine dependence in European Americans.
Dopamine D4 Receptor (DRD4): rs1800955—risk allele C (48bp repeat VNTR)	[165]	Aa analysis of various case-control studies (total 1157 cases vs. 438 controls) found the DRD4 rs1800955 polymorphism associated with cigarette smoking in both heavy and light smokers compared to non-smokers.
Dopamine Transporter Receptor (DAT1): SLC6A3 3′-UTR—risk allele A9 (40bp repeat VNTR)	[166]	DAT1 is a principal regulator of dopaminergic neurotransmission. Lack of literature exists examining the relationship between DAT1 polymorphisms and nicotine dependence. One study saw no association between this particular DAT1 polymorphism and nicotine dependence; however, other studies have seen a strong association with other substances of abuse.
Catechol-O-Methyltransferase (COMT): rs4680—risk allele G (Val158Met)	[167]	COMT is a strong candidate gene that contributes to substance use disorder and schizophrenia. An analysis of 602 nuclear families of African American and European American origin showed an association of COMT rs4680 polymorphism with susceptibility to nicotine dependence that is ethnic- and gender-specific.
µ-Opioid Receptor (OPRM1): rs1799971—risk allele G (A118G)	[168]	Polymorphisms of the OPRM1 gene expressing µ-opioid receptors could be significantly associated with some features of substance dependence. In a meta-analysis of 25 datasets with over 25,000 subjects from European ancestry, results indicated the OPRM1 risk allele G was associated with general substance dependence, including nicotine dependence.
γ-Aminobutyric Acid (GABA) A Receptor, β-3 Subunit (GABRB3): CA repeat—risk allele 181	[169]	Lack of literature exists examining the relationship between GABRB3 and nicotine dependence. However, many studies have examined an association between the GABRB3 polymorphisms related to other substance use disorders, including alcohol dependence. A family-based association analysis suggests GABRB3 may be involved in the risk for alcohol dependence.
Monoamine Oxidase A (MAO-A): 3′ 30bp VNTR—risk allele 4R DNRP	[170]	A clinical study of Japanese outpatients (217 men and 287 women) revealed an association between the MAO-A polymorphisms and nicotine dependence and smoking behavior for both men and women.
Serotonin Transporter Receptor (5HTT) Linked Promoter Region (5HTTLPR) in SLC6A4: rs25531—risk allele S′	[171]	A case-control studied examined the association between the 5-HTTLPR genotype and smoking behavior in Caucasians from Northern Poland (149 cases vs. 158 controls). No significant association was found; however, numerous non-genetic factors may have strongly influenced this genetic susceptibility.

**Table 2:** Prenatal Nicotine Effects on Body Weight in Rodents.

Subjects	ROA	Nicotine Dosage	Length of Exposure	Δ Birth Weight Compared to Controls	Δ Adolescent Body Weight Compared to Controls	References
Female Balb/C mice pups	Sc injection	1.5 mg/kg 2×/day	GD 9–GD 18	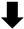 PND21-42	N/A	Zhao et al., 2019 [202]
Male Wistar rat pups	Sc injection	1 mg/kg/day	14 days before mating—PND21	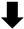	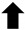 PND49–PND182	Holloway et al., 2005 [192]
Male Wistar rat pups	Sc minipump	6 mg/kg/day	PND2–PND16	N/A	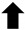 PND180	Conceição et al., 2015 [222]
Male Wistar rat pups	Sc injection	1.0 mg/kg 2×/day	GD9–PND28	No change	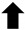 PND28 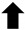 PND84–PND182	Fan et al., 2016 [11]
Female and Male Sprague–Dawley rat pups	Implantable nicotine pellet	0, 15, 25 mg 15mg + 1 and 25mg + 2mg/kg/day	GD0–GD20PND1–PND9	No change	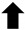 Females PND35- PND91 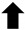 Males on PND35	Chen and Kelly, 2005 [232]
CD1 mice pups	Sc injection	2 mg/kg 2×/day	GD0 to PND0	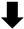	No change	Santiago and Huffman, 2012 [233]
Male Wistar rat pups	Sc injection	1 mg/kg body weight/day	14 days before mating—PND21	No change	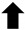 PND70–PND182	Gao et al., 2005 [234]

Abbreviations: Route of administration (ROA), gestational day (GD), postnatal day (PND), subcutaneous (SC), change in (Δ).

**Table 3:** Prenatal Nicotine Exposure Effects on Weight in Humans.

Participants	Age (Years)	Length of Exposure (Months)	Dose (Cigarettes/Day)	Δ in Birthweight from Controls	Δ in Weight/BMI from Controls (Childhood/Adolescence)	Δ in Weight/BMI from Controls (Adult)	References
32,747 children	0–7	0–15 mo	0–20+	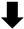	Weight: 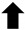 BMI: N/A	Weight: N/A BMI: N/A	Møller et al., 2014 [16]
35,370 daughters	5–18+	0–9 mo	1–25+	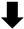	Weight: N/A BMI: 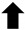	Weight: N/A BMI: 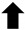	Harris et al., 2013 [178]
266 children	Newborn	0–9 mo	0–29	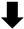	Weight: N/A BMI: N/A	Weight: N/A BMI: N/A	Andersen et al., 2009 [20]
5342 children	0–4	0–9 mo	0–5+	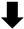	Weight: 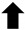 BMI: 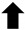	Weight: N/A BMI: N/A	Dürmus et al., 2011 [235]
288 children	8–18	0–9 mo	0–10+	N/A	Weight: N/A BMI: 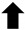	Weight: N/A BMI: 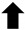	Hill et al., 2005 [236]
5689 children	9.9	0–9 mo	1–20+	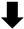	Weight: 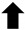 BMI: 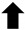	Weight: N/A BMI: N/A	Leary et al., 2006 [237]
912 children	Newborn	0–9 mo	1–30	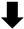	Weight: N/A BMI: N/A	Weight: N/A BMI: N/A	Lewandowska et al., 2020 [227]
174 children	0–2	0–9 mo	0–7	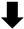	Weight: 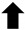 BMI: N/A	Weight: N/A BMI: N/A	Molnar et al., 2017 [18]
18,297 children	Newborn	0–9 mo	1–20+ daily	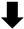	Weight: N/A BMI: N/A	Weight: N/A BMI: N/A	Ward et al., 2007 [226]

Abbreviations: Months (mo), change in (Δ).

## Data Availability

Not applicable.
